# Comment on ‘A Prospective Evaluation of Glucagon Stimulation Test Safety in Adults With Chronic Moderate‐to‐Severe Traumatic Brain Injury’

**DOI:** 10.1002/edm2.70288

**Published:** 2026-07-16

**Authors:** Aisha Zahir, Muhammad Faizan Khan, Gulalai Tariq

**Affiliations:** ^1^ Nowshera Medical College Nowshera Pakistan; ^2^ Khyber Medical University Peshawar Pakistan

To the Editor,

1

We read with interest the study by Snyder et al. [1] investigating the safety of the glucagon stimulation test (GST) in adults with chronic moderate‐to‐severe traumatic brain injury (TBI). The authors provide valuable, reassuring preliminary data regarding favourable short‐term tolerability. However, several methodological considerations may influence the interpretation and generalizability of these findings.

A major limitation is the highly selected study population. Participants were recruited from a specialized neuroendocrine registry and included only individuals who survived at least 1 year post‐injury and were clinically stable. Consequently, patients with greater disability, medical instability, or limited access to tertiary care were likely underrepresented. These findings may therefore not generalize to the broader chronic TBI population, particularly those with severe neurological impairment or higher comorbidity burdens [2].

Furthermore, adverse events were assessed only during a 240‐min observation period, restricting the evaluation to acute tolerability. Delayed complications—such as recurrent hypoglycemia, prolonged gastrointestinal symptoms resulting in dehydration, or delayed cardiovascular events—may have gone uncaptured within this brief window. This is clinically important because chronic TBI patients frequently exhibit underlying autonomic dysfunction and multiple comorbidities that increase vulnerability to delayed adverse effects [3, 4].

Additionally, the absence of a comparator group means acute adverse events cannot be definitively attributed to the GST itself. They could easily stem from background TBI symptomatology, including spontaneous autonomic fluctuations, chronic headache, or nausea, which are common in this population independent of any pharmacological challenge.

Accordingly, the absence of observed harm should not be equated with an absence of risk [5].

While this study offers important preliminary insights into GST tolerability, its findings are not definitive proof of procedural safety. Future multicenter studies involving larger, more diverse populations with extended observation periods are needed. In particular, utilizing simple remote symptom diaries or telephone check‐ins to extend follow‐up 48–72 h beyond the initial test may help identify delayed complications that might otherwise remain undetected.

## Author Contributions

**Muhammad Faizan Khan:** writing – review and editing, supervision. **Aisha Zahir:** writing – original draft, writing – review and editing, conceptualization, methodology, project administration, investigation. **Gulalai Tariq:** writing – review and editing.

## Funding

The authors have nothing to report.

## Ethics Statement

The authors have nothing to report.

## Consent

The authors have nothing to report.

## Conflicts of Interest

The authors declare no conflicts of interest.

## Data Availability

The authors have nothing to report.
